# Flow-cytometric cell sorting coupled with UV mutagenesis for improving pectin lyase expression

**DOI:** 10.3389/fbioe.2023.1251342

**Published:** 2023-08-31

**Authors:** Ke Fang, Jun Ma, Xinyu Wang, Ziting Xu, Ziyang Zhang, Piwu Li, Ruiming Wang, Junqing Wang, Chuying Sun, Ziyang Dong

**Affiliations:** ^1^ State Key Laboratory of Biobased Material and Green Papermaking (LBMP), Qilu University of Technology, Jinan, Shandong, China; ^2^ School of Bioengineering, Qilu University of Technology, Jinan, Shandong, China

**Keywords:** pectin lyase, UV mutagenesis, flow cytometry, *Escherichia coli*, expression

## Abstract

**Introduction:** Alkaline pectin lyase is an important enzyme with a wide range of applications in industrial production, It has been widely used in many important fields such as fruit juice processing and extraction, the dyeing and processing of cotton and linen textiles, degumming plant fibers, environmental industrial wastewater treatment, and pulp and paper production. PGLA-rep4 was previously generated as a modified alkaline pectin lyase with high specific activity at pH 11.0°C and 70°C. However, the pre-constructed high-activity pectin lyase expression strains are still difficult to apply in industrial production due to their limited enzymatic activity. We hope to solve these problems by combining modern breeding techniques with high-throughput equipment to rapidly screen alkaline pectin lyase with higher enzymatic activity and lower cost.

**Methods:** We fused the genes encoding PGLA-rep4 and fluorescent protein *egfp* using a flexible linker peptide and ligated them into a temperature**-**sensitive plasmid, pKD46. The constructed screening plasmid pKD46-PGLA-rep4-*egfp* was then transformed into an expression host and screened via flow-cytometric cell sorting coupled with UV mutagenesis.

**Results:** Following mutagenesis, primary screening, and secondary screening, the high-expression strain, named *Escherichia coli* BL21/1G3, was obtained. The screening plasmid pKD46-PGLA-rep4-*egfp* was eliminated, and the original expression plasmid pET28a-PGLA-rep4 was then retransformed into the mutant strains. After induction and fermentation, pectin lyase activity in *E. coli* BL21/1G3 was significantly increased (1.37-fold relative to that in the parental *E. coli* BL21/PGLA-rep4 strain, *p* < 0.001), and the highest activity was 230, 240 U/mL at 144 h. Genome sequencing revealed that genes encoding ribonuclease E (RNase E) and diadenosine tetraphosphatase (ApaH) of *E. coli* BL21/1G3 were mutated compared to the sequence in the original *E. coli* BL21 (DE3) strain, which could be associated with increased enzyme expression.

**Discussion:** Our work provides an effective method for the construction of strains expressing pectin lyase at high levels.

## 1 Introduction

During pectin degradation, the function of pectin lyase is to break the α-1,4 glycosidic bonds of pectin polymers via a trans-elimination reaction, which generates double bonds and reducing groups, resulting in the formation of Δ4,5-unsaturated oligogalacturonide products (Yadav et al., 2017; Ahmed et al., 2021; Patel et al., 2022). In nature, about 50% of pectinolytic enzymes come from fungi, 35% from bacteria, and the remaining 15% from animals and plants (Demir et al., 2014; Shrestha et al., 2021). The main bacterial species and genera able to produce pectin lyase are *Bacillus*, *Erwinia*, *Penicillium*, and *Aspergillus* (Klug-Santner et al., 2006; Ribeiro et al., 2010; Abd El-Rahim et al., 2020; Yang et al., 2020).

Pectin lyase is a highly valuable industrial enzyme that has been widely used in various important fields, such as fruit juice processing and extraction, cotton and linen textile dyeing and processing, plant fiber degumming, environmental industrial wastewater treatment, and pulp and paper production (Bruhlmann et al., 2000; Phugare et al., 2011; Zhang et al., 2019; Jaworska et al., 2022). As more attention is given to the environment, reducing pollution and energy consumption has become an urgent issue in traditional industries. Pectin lyase has the advantages of environmental friendliness and low energy-consumption requirements, and its market demand continues to grow steadily owing to its important application value.

Currently, mutation breeding methods are widely used to enhance enzyme expression, and UV mutagenesis is one of the most common traditional breeding techniques. When used for the selection of microbial strains, it can improve the yield of extracellular products; moreover, it is a simple, fast, and efficient method. Wang et al. (2014) screened a mutant strain, JNFE1126, with high nattokinase production using UV combined with the ^60^Co-γ-ray-mediated mutagenesis of *Bacillus subtilis*, and their results showed that the nattokinase production of the mutant strain was 2-fold higher than that of the wild-type strain. Moreover, through the mutagenesis of *Saccharopolyspora erythraea* using UV light and selection of tylosin-resistant mutants, Fallahpour et al. (2017) obtained a mutant whose erythromycin production was 20-fold higher than that of the wild-type strain. The screening and isolation of highly expressed strains after mutagenesis are labor intensive; therefore, high-throughput methods incorporating the screening of high-expression strains are also needed.

Fluorescence-activated cell sorting (FACS) is a flow cytometry-based technique used for efficient single-cell sorting. It requires the coupling of fluorescent products to encoded proteins to establish a detection pathway for fluorescent signals (Copp et al., 2014; Markel et al., 2020; Vitelli et al., 2021). The sorting efficiency of sorting flow cytometers can reach tens of thousands of cells per second with high sorting accuracy, which makes them a powerful tool for high-throughput screening strategies. Sielatycka et al. (2021) used nucleic acid fluorescent dyes based on cell membrane integrity to assess *Lactobacillus* and *Bifidobacterium* in lyophilized powders and found that in the studied probiotic products, results could be available within as little as 2 h using flow cytometry, much shorter than the 96 h required for plate counting. With the help of high-throughput screening equipment, mutant strains with high expression levels can be rapidly sorted. This method combines accuracy and sensitivity and can be applied to a wide range of microbial cell types.

**FIGURE 1:**
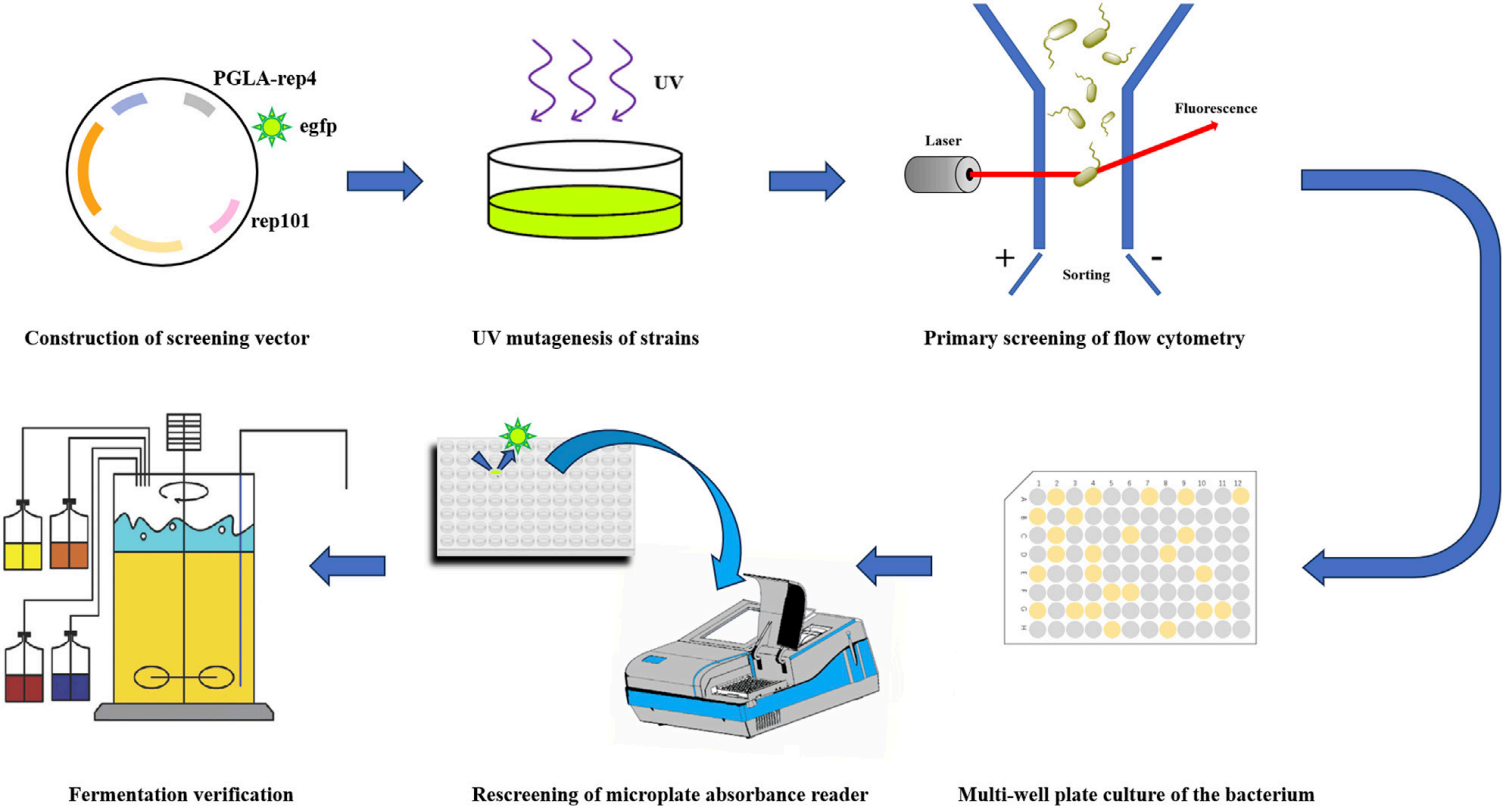
A technical roadmap for flow cytometric sorting combined with UV mutagenesis to improve the expression of pectin lysozyme.

In a previous study, we obtained a pectin lyase, PGLA-rep4, with the same temperature and alkalinity tolerance but 1.13-fold higher enzyme activity, compared to those of the wild-type, using a rational design method and by replacing the unstable region in the gene sequence based on a fragment substitution technique. Its enzyme activity was found to reach 168,058 U/mL, with an optimum pH of 11.0 and optimum temperature of 70°C (Li et al., 2022). PGLA-rep4 has a wide range of applications in industry, and we hope to combine modern breeding techniques with high-throughput equipment to further improve enzyme activity, thereby minimizing the reaction time and increasing substrate conversion. In this study, we fused the gene encoding PGLA-rep4 with that encoding the fluorescent protein *egfp* using a flexible linker peptide and ligated them into the temperature-sensitive plasmid pKD46. The constructed screening plasmid, pKD46-PGLA-rep4-*egfp*, was then transformed into expression hosts and screened via flow cytometry and UV mutagenesis (Figure 1).

## 2 Materials and methods

### 2.1 Strains and culture media

Luria–Bertani (LB) liquid medium with or without ampicillin (100 μg/mL) or kanamycin (50 μg/mL) was used for the growth and transformation of *Escherichia coli* at 37°C with 200 rpm of shaking, and LB solid medium (containing 2% agar) was used for screening at 37°C. Yeast Extract-Peptone-Dextrose (YPD) liquid medium with or without G418 (100 μg/mL) was used for the growth and transformation of yeast at 30°C with 200 rpm of shaking, and screening was performed using YPD solid medium (containing 2% agar) incubated at 30°C. Recombinant *E. coli* containing a temperature-sensitive replicon was grown and transformed using LB liquid medium containing ampicillin (100 μg/mL) at 30°C with 200 rpm of shaking and screened using LB solid medium (containing 2% agar), incubated at 30°C. The fermentation of recombinant yeast strains to induce protein expression was performed under shake flask conditions using Buffered Minimal Glycerol-complex Medium (BMGY) and Buffered Methanol-complex Medium (BMMY) media. The BMGY medium contained the following: 10 g/L glycerol, 10 g/L yeast extract, 20 g/L peptone, 13.4 g/L yeast nitrogen base (YNB), 4 × 10^−4^ g/L biotin. The BMMY medium contained the following: 10 g/L yeast extract, 20 g/L peptone, 13.4 g/L YNB, 4 × 10^−4^ g/L biotin. All strains and plasmids used in this study are listed in Table 1, and all primers used in this study are listed in Table 2.

**TABLE 1:** Strains and plasmids used in this study.

Strain/plasmid	Relevant genotype/description	Source
Strains		
*E. coli* BL21 (DE3)	F- ompT hsdS(rB - mB -) gal dcm(DE3)	Vazyme Biotech
*Pichia pastoris* GS115		Thermo Fisher Scientific
*E. coli* BL21/PGLA-rep4	Expression vector of *E. coli* BL21, for expression of PGLA-rep4 gene	This study
*P*. *pastoris* GS115/PGLA-rep4	Expression vector of *P. pastoris* GS115, for expression of PGLA-rep4 gene	This study
*E. coli* BL21/1G3	Screened mutant strains with the highest enzyme activity	This study
*E. coli* BL21/E	Expression vector of *E. coli* BL21, connecting *egfp* fluorescent protein for screening	This study
*E. coli* BL21/1H1	Screened mutant strains with high enzyme activity	This study
*E. coli* BL21/1G4	Screened mutant strains with high enzyme activity	This study
*E. coli* BL21/3E5	Screened mutant strains with high enzyme activity	This study
*E. coli* BL21/3F7	Screened mutant strains with high enzyme activity	This study
Plasmids/fragments		
pET28a-PGLA-rep4	Expression vector of *E. coli* BL21, induced by IPTG	Vazyme Biotech
pPIC9K-PGLA-rep4	Expression vector of *P. pastoris* GS115, induced by methanol	GenScript
pUC57-*egfp*	Cloning vector of *E. coli* BL21, connecting fluorescent protein *egfp*	GenScript
pKD46	RED recombinant system plasmid vector of *E. coli* BL21, connecting temperature-sensitive replicas	GenScript
pKD46-PGLA-rep4	Connecting temperature-sensitive replicas, constructed for screening	This study
pKD46-PGLA-rep4-*egfp*	Connecting temperature-sensitive replicas and *egfp*, constructed for screening	This study

**TABLE 2:** Primers used in this study.

Primers	Sequence(5′-3′)
kd-F	AGG​AGG​AAC​TAT​ATC​CGG​ATC​CAT​GGG​TAT​GGA​C
kd-R	TTA​TCA​TGC​AAC​TCG​TAG​GAG​ATA​TCG​CCA​GCG​TCG​CA
et-F	TGT​GCG​ACG​CTG​GCG​ATA​TCT​CCT​ACG​AGT​TGC​AT
et-R	ATA​TCC​GGA​TCC​ATG​GGT​ATG​GAC​AGT​TTT
egf-F	TCG​CCG​CTC​GAG​AGC​GGT​GGT​GGT​TCA​GGT
egf-R	GTG​GTG​GTG​GTG​CTC​GAG​TTT​ATA​CAG​TTC​ATC​CAT​A
qPGLA-F	GCG​AAC​GTG​AAC​TTT​TCC​ATG
qPGLA-R	TTC​CAA​GAA​TCG​TGC​ACG​TAA​T
gapA-F	GTG​CGA​AGA​AAG​TGG​TTA​TGA​C
gapA-R	GTA​GCG​GTA​GTA​GCG​TGA​AC

### 2.2 Pectin lyase heterologous expression vector construction

In a previous study, we obtained the pectin lyase PGLA-rep4, which showed a 1.13-fold increase in enzymatic activity compared to that of the wild-type, using a rational design, with a fragment substitution technique to replace the unstable region in the gene sequence. To verify the difference in enzymatic activity in different hosts, PGLA-rep4 was ligated into pET28a and pPIC9K vectors and transformed into *E. coli* BL21(DE3) and *P. pastoris* GS115 to obtain heterologous expression strains, specifically *E. coli* BL21/PGLA-rep4 and *Pichia pastoris* GS115/PGLA-rep4, which were then assayed for enzyme activity.

### 2.3 Expression and enzyme activity analysis using recombinant strains

The *AOX1* promoter in *P. pastoris* is strictly induced by methanol to transcriptionally regulate the expression of related genes, and BMMY is more suitable for propagation of the expression vector pPIC9K, in conjunction with BMGY. After activation, *P. pastoris* GS115/PGLA-rep4 was transferred to 50 mL of BMMY medium based on a 1% inoculum and incubated overnight in shaking flasks. The BMGY was centrifuged at 3,000 rpm for 10 min, the supernatant was discarded, and the precipitate were resuspended in 50 mL of BMMY medium and then incubated at 30°C with 200 rpm of shaking for 72 h. The concentration of methanol was maintained at 1% by replenishing it every 24 h to induce protein expression. Meanwhile, *E. coli* BL21/PGLA-rep4 was transferred to 50 mL of LB medium based on a 1% inoculum, when the density of the bacteria reached approximately 0.7 OD_600_, after which isopropyl β-D thiogalactoside (IPTG) at a final concentration of 0.5 mM was added, and cells were incubated at 25°C for 12 h to induce protein expression.

A comparison of different host bacteria was performed based on an alkaline pectin lyase enzymatic activity assay. The supernatant was removed by centrifuging 30 mL of fermentation broth at 12,000 rpm for 10 min. The precipitate was then resuspended in 8 mL of PBS (pH 7.4) and sonicated for 20 min, for *E. coli*, or 40 min, for *P. pastoris*, before centrifugation at 12,000 rpm for 10 min. Then, the supernatant was collected as the desired crude enzyme solution. The supernatant of the culture after the induced expression was centrifuged as the crude enzyme solution, and a 25 mL colorimetric tube was used, to which 1.9 mL of glycine-sodium hydroxide buffer (pH 11.0) containing 0.2% pectin substrate was added, followed by 100 µL of the appropriately diluted crude enzyme solution. After mixing, the reaction was carried out at 70°C for 10 min. Finally, 3 mL of 0.03 M phosphoric acid solution was added to terminate the reaction, and the absorbance value of the unsaturated product at 235 nm was measured using a UV spectrophotometer; enzyme activity was determined according to the calculation formula(Yadav et al., 2008; Cerreti et al., 2017; Zhou et al., 2017).

### 2.4 Determination of PGLA-rep4 hydrolysis products

The reaction with PGLA-rep4 was carried out by adding 0.2% pectin in Gly-NaOH buffer (pH 11.0) for 10 min at 70°C. The reaction was then terminated by adding 3 mL of 0.03 M phosphoric acid. The hydrolysates released from pectin were evaluated by performing high-performance liquid chromatography (HPLC). The resulting sugars were separated on a Hi-Plex Ca column (300 mm × 7.7 mm; Agilent, Santa Clara, CA, United States) using a mobile phase of pure water at a flow rate of 0.6 mL/min. The column temperature was maintained at 80°C, and the sample was injected into 10 μL. The peak area of the sugar was measured using a Shimadzu RID-10A refractive index detector. The peak times of the hydrolysate and corresponding standards were compared to determine the type of hydrolysate.

### 2.5 Construction of temperature-sensitive screening plasmid for *E. coli*

pKD46 is a temperature-sensitive plasmid that relies on the rep101 replication protein; it can replicate normally at 30°C and is essentially unreplicable at 37°C, and it can be utilized to rapidly eliminate the enhanced green fluorescent protein (*egfp*) gene in mutant strains (Liao et al., 2018). The PCR reaction was performed with the pKD46 plasmid as the template and kd-F and kd-R as primers; here, DNA was pre-denatured at 95°C for 3 min, denatured at 95°C for 15 s, annealed at 60°C for 30 s, pre-extended at 72°C for 90 s, cycled 30 times, then extended at 72°C for 5 min, and finally kept at 4°C. A temperature-sensitive fragment, of 3,285 bp in length, was obtained via gel purification of the replicon. The PCR reaction was then performed with the fragment containing the PGLA-rep4-encoding gene, T7 promoter, and *lac*Ⅰ gene with the pET28a-PGLA-rep4 plasmid as the template, using et-F and et-R as primers. Here, the DNA was pre-denatured at 95°C for 3 min, denatured at 95°C for 15 s, annealed at 60°C for 30 s, and pre-extended at 72°C for 90 s, cycled 30 times, and then, extension was performed at 72°C for 5 min; the sample was finally kept at 4°C. The fragment containing the PGLA-rep4-encoding gene, T7 promoter, and *lacⅠ* gene, with a length of 2,834 bp, was obtained via gel purification. The obtained gene fragment was ligated using a seamless cloning technique to finally obtain the recombinant plasmid pKD46-PGLA-rep4.

The pET28a-pKD46-PGLA-rep4 plasmid was then single endonuclease digestion to obtain the linearized vector. The green fluorescent protein vector pUC57-*egfp* was provided by GenScript, and the PCR reaction was performed with this plasmid as the template using egf-F and egf-R as primers. Here, the DNA was pre-denatured at 95°C for 3 min, denatured at 95°C for 15 s, annealed at 60°C for 30 s, and pre-extended at 72°C for 90 s, cycled 30 times, and then, extension was performed for 5 min, and the sample was finally kept at 4°C. The green fluorescent protein *egfp*-encoding gene fragment, with a length of 783 bp, was obtained via gel purification. The obtained linearized pKD46-PGLA-rep4 vector and *egfp* gene fragment were ligated together using a seamless cloning technique to finally obtain the recombinant plasmid pKD46-PGLA-rep4-*egfp*. Next, the recombinant plasmid was introduced into *E. coli* BL21 (DE3), which was cultured in LB containing ampicillin (100 μg/mL) and on LB agar plates; after successful incubation, single colonies were selected and grown in LB liquid medium containing ampicillin (100 μg/mL).

### 2.6 Mutagenesis conditions for the recombinant strains

To determine the optimal conditions for UV mutagenesis, 3 mL of a diluted *E. coli* BL21/E bacterial solution (1.62 × 10^7^ CFU/mL) was placed in a disposable Petri dish, and the suspension was irradiated at a distance of 28 cm from a 36 W UV lamp in the dark for 0, 15, 30, 45, 60, 70, and 90 s. After irradiation, the suspension was diluted to 10^–5^, 10^–6^, and 10^–7^, incubated on LB solid plates and observed. After irradiation, the suspension was diluted to 10^–5^, 10^–6^, and 10^–7^ and then incubated on LB solid plates, and the number of colonies was enumerated to calculate the cell death. The time at which cell death reached >90% was regarded the best time for mutagenesis, as it corresponds to the highest probability of a strain undergoing forward mutation (Kawai et al., 2022).

### 2.7 High-throughput screening of mutant strains

The fermentation broth was transferred to 50 mL of LB medium after UV irradiation and grown at 30°C with 200 rpm of shaking until the OD_600_ was approximately 0.7. IPTG, at a final concentration of 0.5 mM, was added for induction at 25°C for 12 h. The fermentation broth was centrifuged at 5,000 rpm for 10 min, and then, the supernatant was discarded and the precipitate was washed with 0.9% saline twice. Then, the supernatant was discarded, the precipitate was rinsed 2–3 times with 0.9% saline, and finally, the precipitate was resuspended in saline. The resuspended cytosol was diluted 100-fold and then sorted by performing flow cytometry. Sodium chloride solution, at a concentration of 0.85%, was used as the flow cytometer sheath solution, and fluorescent microspheres were mixed for quality control, with respect to the flow cytometer optical path and liquid flow system. The prepared samples were sorted via flow cytometric analysis using a Moflo XDP model ultra-fast flow cytometer (Beckman Coulter) equipped with a trichrome laser. The maximum excitation wavelength of the green fluorescent protein *egfp* is approximately 488 nm, and the maximum emission wavelength is approximately 510 nm; therefore, a 485 nm laser was used to efficiently achieve excitation of the fluorescent protein, and the FL 1 channel of the experimental model flow cytometer was selected to determine the emission wavelength. The injector differential pressure was adjusted such that the program was executed at a system pressure of 60 Psi, while keeping the sample pressure 0.1–0.3 Psi higher than the sheath fluid pressure, to ensure the detection of approximately 10,000 target cells per second. Several single cells with uniform size and strong fluorescence were selected, and their fluorescence intensity was measured using a microplate reader after induction.

### 2.8 Genome analysis of mutant bacteria

Tri-generation PacBio sequencing technology was combined with Illumina sequencing technology to perform genome sequencing analysis of the high pectin lyase-producing strain *E. coli* BL21/1G3, obtained via UV mutagenesis. The starting strain (accession number: NZ_CP053601) and the mutant strain were compared, and the mutated sequences were compared with the genome sequence of *E. coli* BL21 obtained using BLAST in the NCBI database to obtain information related to the increased expression of pectin lyase in the mutant strain.

### 2.9 Analysis of mRNA abundance via qPCR

After expression was induced in the strain, mRNA was extracted using the Total RNA Extraction Kit (Solarbio). qPCR-specific primers were designed based on the first 500 bases of PGLA-rep4, and a pair of primers was designed by selecting an *E. coli* reference gene (*gapA*) as an internal reference (Table 2). qPCR reactions (20 μL) contained 10 μL of SsoAdvanced Universal SYBR Green Supermix (BioRad), 20 μM primers, 10 ng of mRNA extract, and DEPC water. Reactions were measured using a LightCycler 96 Detection System (Roche) with the following thermocycling parameters: one cycle at 95°C for 2 min; 40 cycles at 95°C for 10 s, 59°C, for 20 s, and 72°C for 20 s; and final melting curve analysis (59°C–95°C based on 0.5°C/5 s increments).

The effect of the mutation on transcription efficiency was illustrated by measuring the relative abundance of mRNA in the unmutated strain *E. coli* BL21/PGLA-rep4 and the mutated strain *E. coli* BL21/1G3. In qPCR experiments, the mRNA relative abundance in all samples was calculated using the 2^−ΔΔCT^ method. All measurements shown in the text and figures are based on three independent biological replicates.

### 2.10 Statistical analysis

Statistical analysis was performed using IBM SPSS Statistics 29 (IBM SPSS, Turkey). One-way ANOVA was used to analyze the significance of mean differences among samples. The significance of differences was estimated with a 0.05 level of confidence.

## 3 Results

### 3.1 Construction of pectin lyase expression vector and host optimization

**FIGURE 2:**
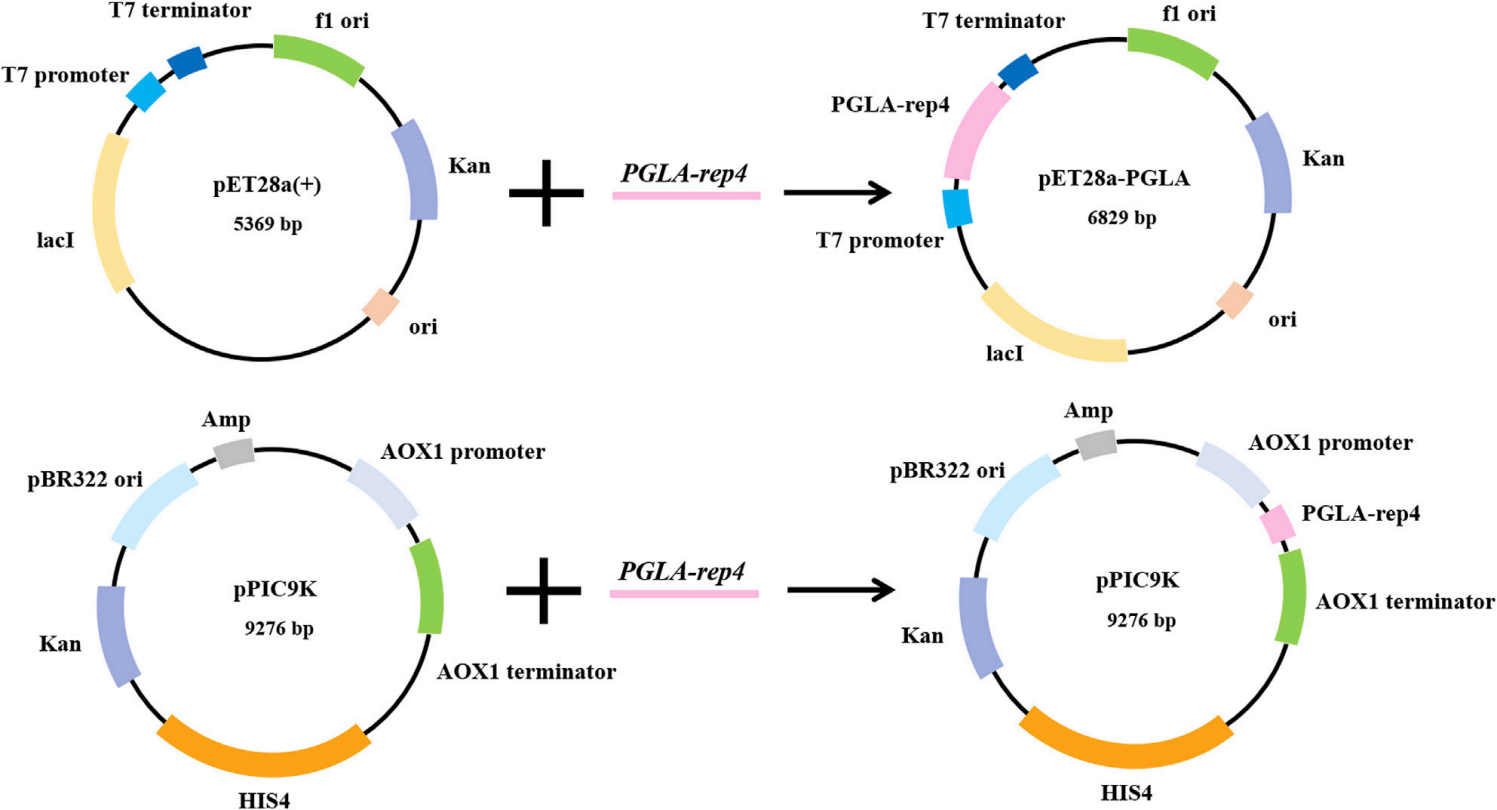
Construction of PGLA-rep4 expression vectors for *Escherichia coli* and *Pichia pastoris*. Two recombinant vectors were constructed, in which pET28a-PGLA-rep4 regulates the transcription of the gene through the T7 promoter and T7 terminator, while pPIC9K-PGLA-rep4 regulates the transcription of the gene through the *AOX1* promoter and *AOX1* terminator.

**FIGURE 3:**
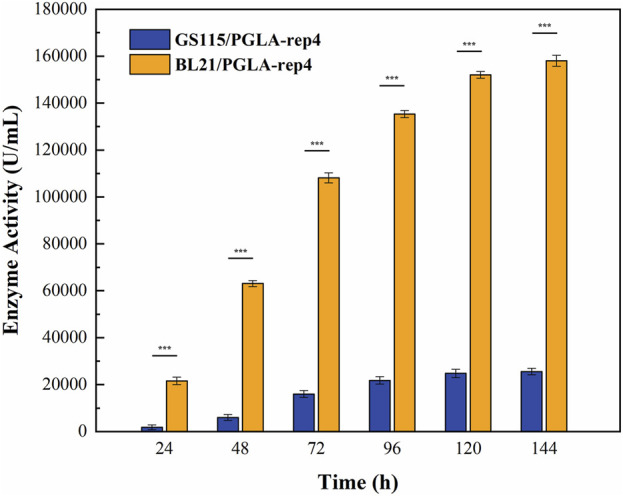
Changes in enzyme activity of PGLA-rep4 *Escherichia coli* expression hosts and *Pichia pastoris* expression hosts under 30 L fermenter conditions. Error bars represent standard deviations.

The host for plasmid expression affects the intensity of enzyme activity. To study the effect of different hosts on pectin lyase expression, plasmids pET28a-PGLA-rep4 and pPIC9K-PGLA-rep4 were constructed (Figure 2; Supplementary Figures S1A-B) and transformed into the host strains *E. coli* BL21(DE3) and *P. pastoris* GS115. After the induction of expression in *P. pastoris* GS115/PGLA-rep4 and *E. coli* BL21/PGLA-rep4, the fermentation broth was centrifuged, resuspended in PBS, and subjected to ultrasonic fragmentation, and the supernatant was centrifuged to determine the enzyme activity. One unit of enzyme activity was defined as the amount of enzyme required to produce the equivalent of 1 μg of unsaturated oligogalacturonan material per minute from the cleavage of polygalacturonan. To verify that PGLA-rep4 was successfully expressed in *E. coli* and *P. pastoris*, we performed SDS-PAGE analysis, and the results proved that PGLA-rep4 was successfully expressed in *E. coli* (Supplementary Figure S2A) and *P. pastoris* (Supplementary Figure S2B), with clear and obvious protein bands and a molecular weight of approximately 35 kDa. After the PGLA-rep4 reacted with the pectin substrate at 70°C for 10 min, galacturonic acid was detected as the main hydrolysis product obtained from fructose using HPLC analysis, whereas pectin was barely detectable (Supplementary Figure S3). The changes in enzyme activity of the recombinant strain under 30 L fermenter conditions were determined and are shown in Figure 3. With the *E. coli* vector, PGLA-rep4 exhibited an activity of 168,058 U/mL, whereas, with the *P. pastoris* GS115 vector, it was only 26,990 U/mL, which shows that *E. coli* is a more suitable host for the expression of PGLA-rep4 than *P. pastoris* GS115. Therefore, *E. coli* BL21(DE3) was chosen as the host bacterium for PGLA-rep4 expression.

### 3.2 Construction of PGLA screening vector

**FIGURE 4:**
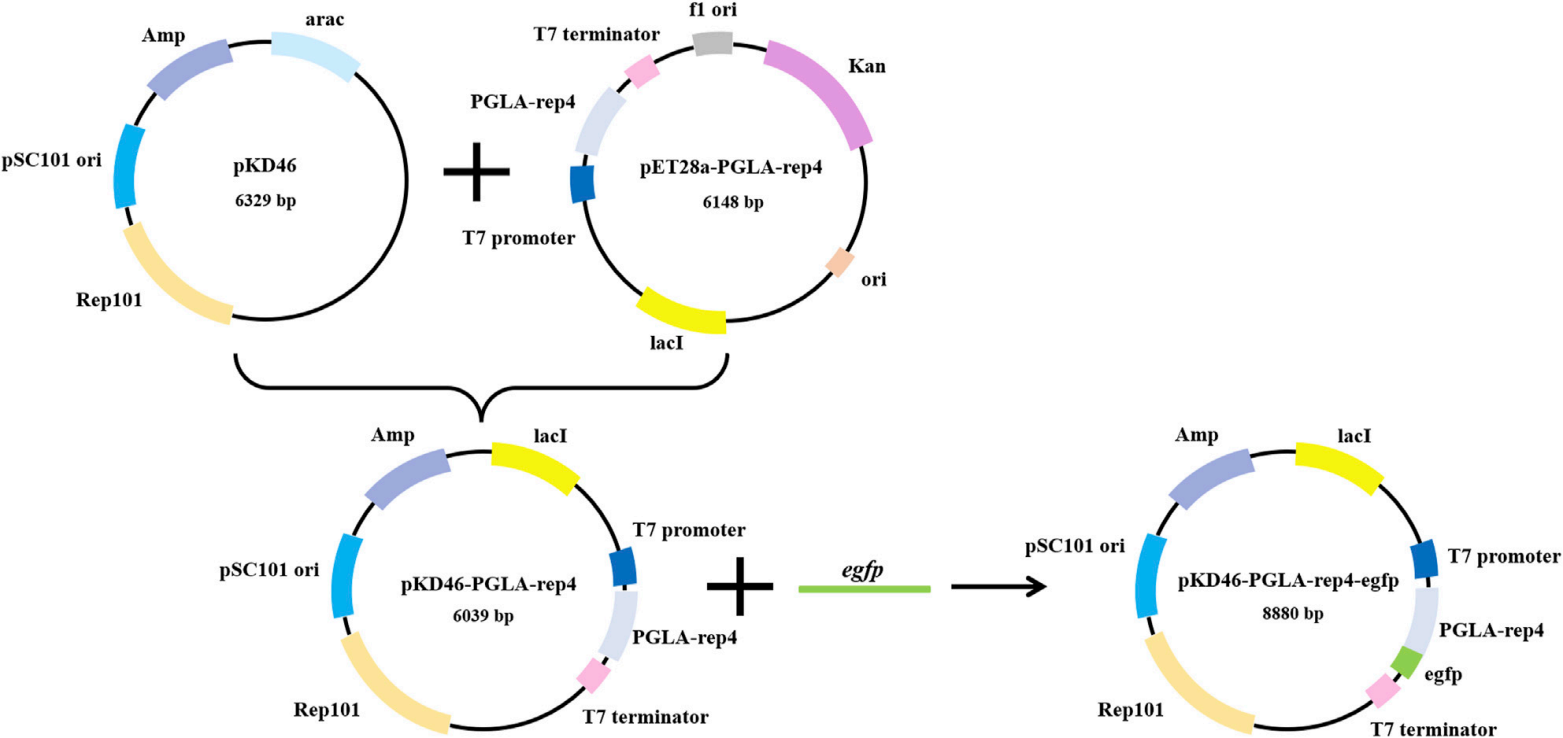
Plasmid integration map of pET28a-pKD46-PGLA-rep4-*egfp*, which contains the rep101 temperature-sensitive protein as well as the *egfp* and PGLA-rep4 genes regulated by the T7 promoter and T7 terminator. The fluorescence intensity of *egfp*, which is linked to PGLA-rep4, becomes higher when its expression is increased, corresponding to a similar increase in the expression of PGLA-rep4.

The UV mutagenesis vector *E. coli* BL21(DE3)/pET28a-pKD46-PGLA-rep4-*egfp* was constructed (Figure 4; Supplementary Figures S4A-D). By linking the enzyme to a fluorescent protein and coupling the expression of this enzyme to the fluorescence intensity, screening *E. coli* mutant hosts that are more favorable for enzyme expression after mutagenesis becomes possible. The fluorescence intensity of the fluorescent protein *egfp* was calculated to determine enzyme activity, and the inserted temperature-sensitive replicon was used to directly avoid the effect of fluorescent protein expression on enzyme activity. Agarose gel electrophoresis showed that the target band was consistent with the size of the target gene, and the sequencing results indicated that the base sequence was complete.

### 3.3 Analysis of cell death of strains under mutagenic conditions

**FIGURE 5:**
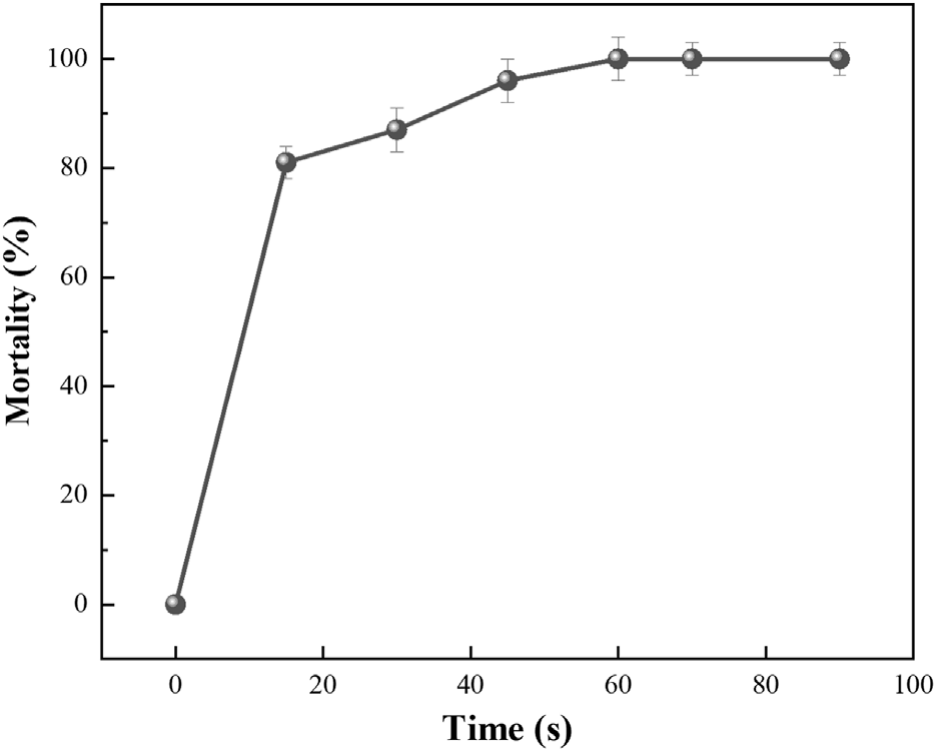
The lethality rate curves of UV mutagenesis for 15, 30, 45, 60, and 70 s, respectively. Error bars represent standard deviations.

The recombinant strain *E. coli* BL21/E was UV-irradiated using a 36 W UV lamp a cell death curve was plotted, as shown in Figure 5. When the UV irradiation time reached 15 s, the lethality of the strain reached about 81%; when irradiated for 30 s, the lethality of the strain reached about 83%; and when irradiated for 45 s, the lethality of the strain reached about 97%. We choose UV irradiation for 35 s as the best mutagenesis time.

### 3.4 Flow cytometric screening of high-yield mutant strains

**FIGURE 6:**
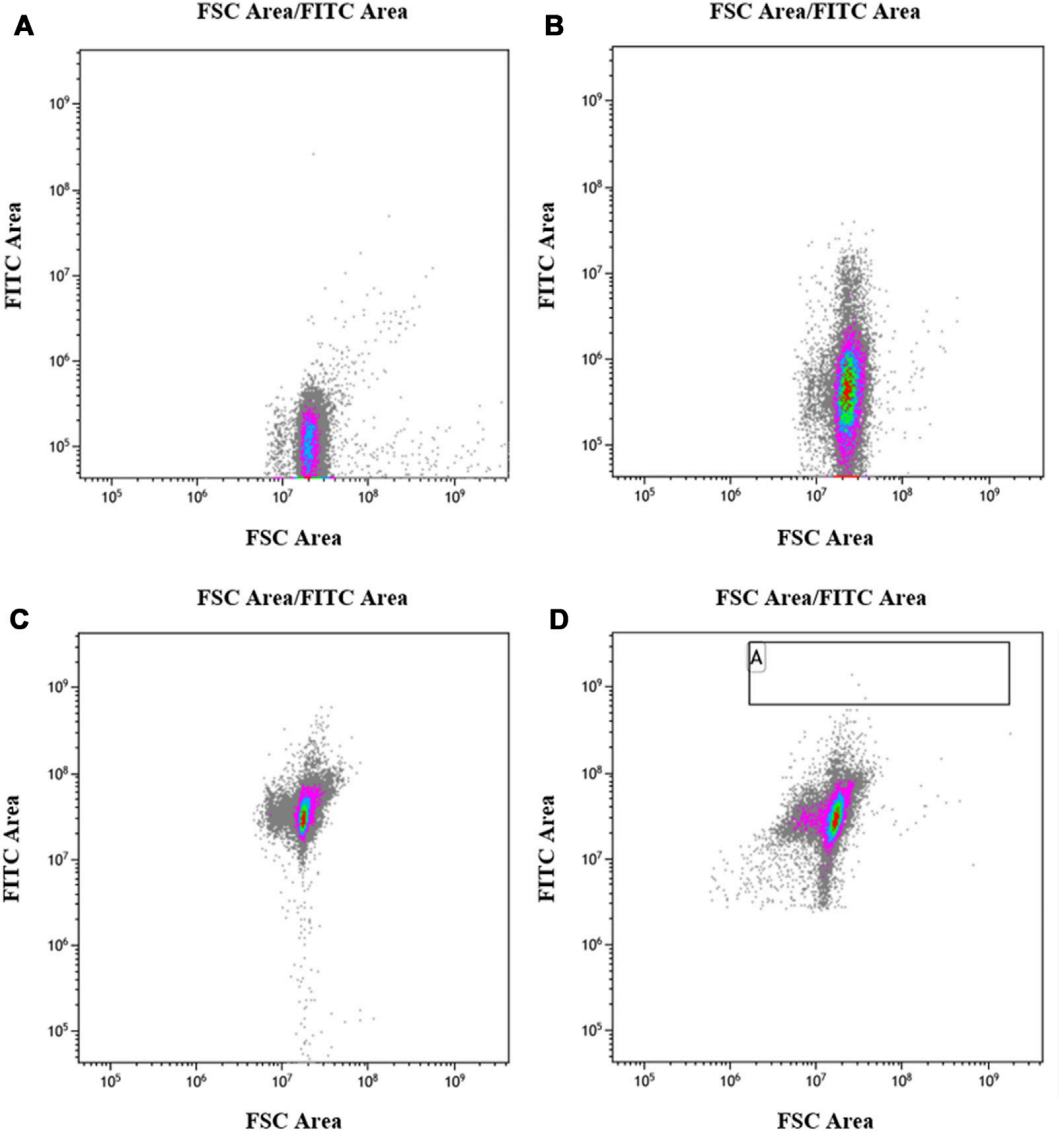
Flow cytometry screening after mutation. **(A)**: *Escherichia coli* BL21 (DE3) control strain without *egfp*; **(B)**: *E. coli* BL21/E strain, containing *egfp*, not induced by IPTG. **(C)**: *E. coli* BL21/E strain, containing *egfp*, induced by IPTG; **(D)**: *E. coli* BL21/E strain after UV mutagenesis, induced by IPTG.

The UV-irradiated bacterial solution was subjected to primary screening using flow cytometry to screen cells of uniform size with strong fluorescence, and the range of the screening area is shown in Figures 6A–D. Figure 6A shows that the *E. coli* strain without the *egfp* gene did not produce fluorescence, whereas Figure 6B shows that the *E. coli* strain containing the *egfp* gene exhibited low fluorescence intensity without induction. Figure 6C shows that the *E. coli* strain containing the *egfp* gene exhibited improved fluorescence intensity after induction, and Figure 6D shows that the fluorescence intensity of most *E. coli* strains containing the *egfp* gene decreased after 35 s of UV irradiation, indicating a reverse mutation. Meanwhile, a small number of strains showed an increase in fluorescence intensity, indicating a forward mutation. The strains with increased fluorescence intensity in the A region, in Figure 6D, were screened, accounting for approximately 1 in 20,000 isolates.

**FIGURE 7:**
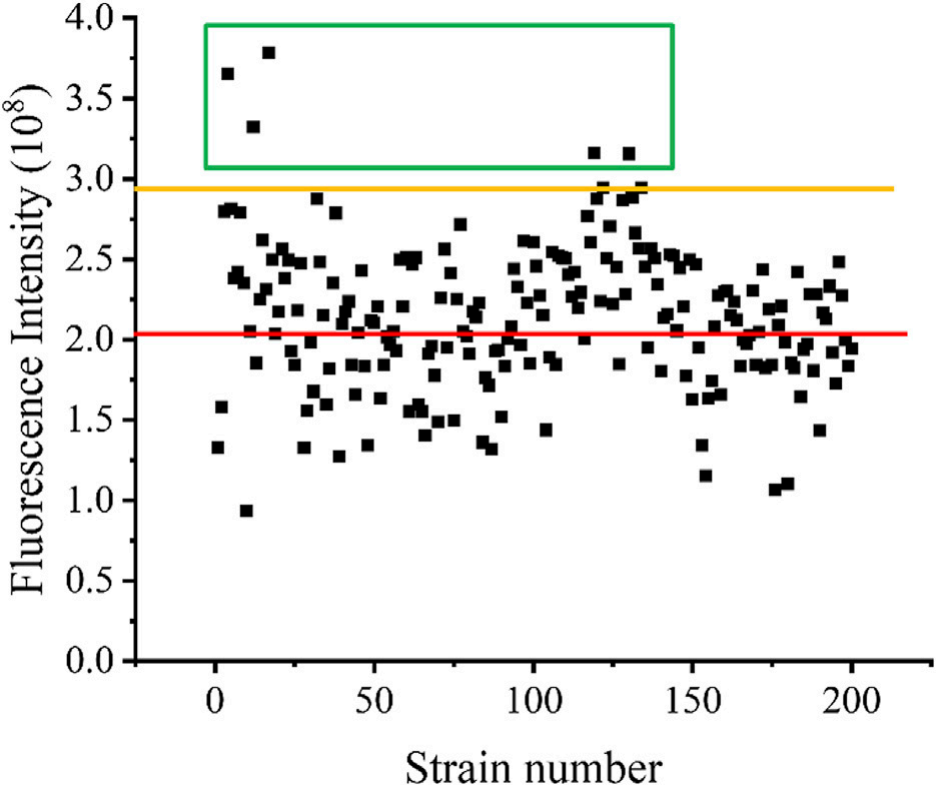
Fluorescence intensity of each mutant strain. The red line represents the fluorescence intensity of strains that have not undergone UV mutagenesis, while the orange line represents the fluorescence intensity of strains that have undergone UV mutagenesis.

**FIGURE 8:**
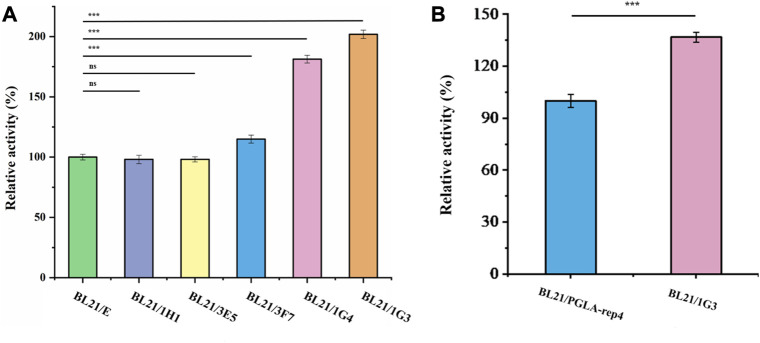
Comparison of enzyme activities of each strain after mutagenesis. **(A)**: Comparison of enzyme activity of each mutant strain after UV mutagenesis. **(B)**: Comparison of the strains exhibiting the highest enzyme activity after mutagenesis with the original strains. Error bars represent standard deviations.

After screening, the fluorescence intensity of each single cell was measured, after growth, multiplication, and expression induction, and the results are shown in Figure 7. The red line indicates the fluorescence intensity produced by the strain without UV irradiation, whereas the orange line represents the fluorescence intensity produced by the strain with UV irradiation. The cells of the five strains for which fluorescence intensity was higher than the highest level of fluorescence intensity of the unmutated strains, in the green box line, were selected and cultured in shake flasks, and their enzyme activity was determined after expression induction. The plasmid of the mutant strain also expressed the fluorescent protein *egfp*, for which the encoding gene was ligated to the plasmid after the pectin lyase gene sequence. Since this could affect the expression of the enzyme, as well as enzyme activity, additional fluorescent strains obtained via mutagenesis were prepared in competent cells by curing the plasmid at 37°C, and the pET28a-PGLA-rep4 plasmid was transformed into each receptor cell. Then, the enzyme activity was measured after culture and inducing expression. As shown in Figure 8A, among the five mutant strains, *E. coli* BL21/1H1, *E. coli* BL21/1G4, *E. coli* BL21/3E5, and *E. coli* BL21/3F7 exhibited 0.98- (ns), 1.81- (*p* < 0.001), 0.98- (*p* < 0.05), and 1.15- (*p* < 0.001) fold the enzyme activity of the starting strain, whereas *E. coli* BL21/1G3 exhibited the highest enzyme activity, which was 2.02- (*p* < 0.001) fold higher than that of the *E. coli* BL21/E strain before UV irradiation. Further, this was approximately 1.37- (*p* < 0.001) fold higher than that of the parental strain *E. coli* BL21/PGLA-rep4 (Figure 8B).

### 3.5 Fermentation of mutant strains and determination of enzyme activity

**FIGURE 9:**
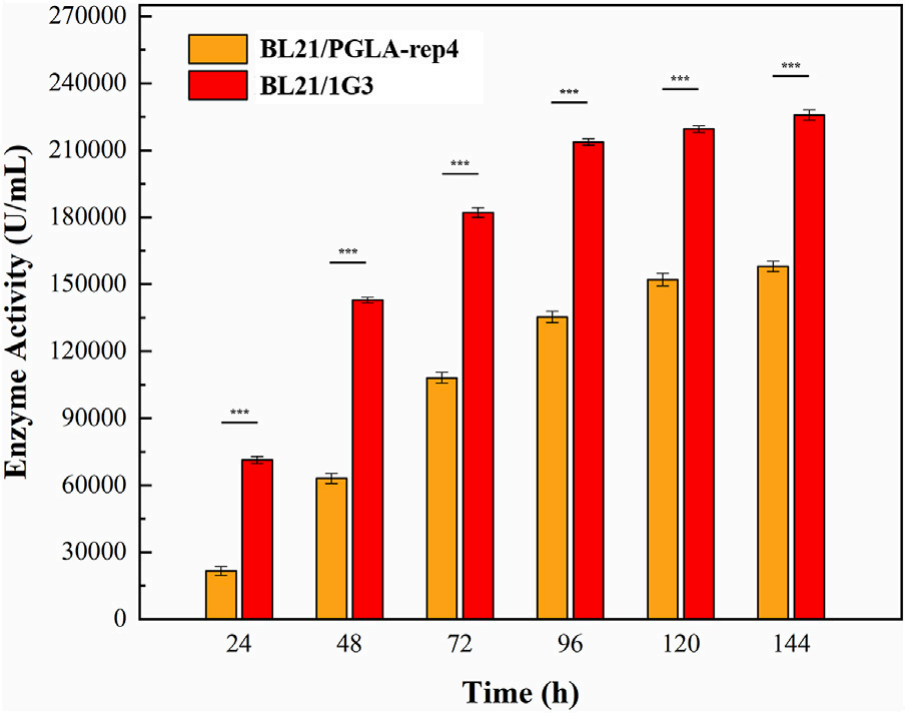
Changes in PGLA-rep4 enzyme activity between mutant and non-mutant strains under 30 L fermenter conditions. Error bars represent standard deviations.

After the induction of expression in *E. coli* BL21/PGLA-rep4 and *E. coli* BL21/1G3, the fermentation broth was centrifuged and resuspended in PBS and subjected to ultrasonic fragmentation. Moreover, the supernatant was centrifuged to determine the enzyme activity. One unit of enzyme activity was defined as the amount of enzyme required to produce the equivalent of 1 μg of unsaturated oligogalacturonan material per minute from the cleavage of polygalacturonan. Changes in the enzyme activity of the recombinant strain under 30 L fermenter conditions are shown in Figure 9. In *E. coli* BL21/PGLA-rep4, the enzyme activity reached 168,058 U/mL, whereas in the mutagenized strain *E. coli* BL21/1G3, the enzyme activity reached 230,240 U/mL, which was approximately 1.37- (*p* < 0.001) fold higher than that in the original strain. This indicated that UV mutagenesis combined with the flow cytometric screening strategy successfully improved the enzymatic activity of PGLA-rep4.

### 3.6 Genome sequence alignment analysis of mutant strains

The genome spliced sequence was compared with the genome sequence of *E. coli* BL21 (DE3), obtained using BLAST from the NCBI database, and one base mutation and one base deletion were identified. The sequencing results have been uploaded to the NCBI SRA database (Registry number: PRJNA988841). Regarding the base mutation, the amino acid was mutated from arginine to leucine, and the corresponding gene was found to encode a ApaH, as determined using BLAST from the NCBI database (Supplementary Figure S5). Regarding the deletion, two bases were missing, and the use of BLAST from the NCBI database showed that the mutated gene encodes RNase E (Supplementary Figure S6). It is thus possible that the mutations in these two enzymes resulted in the increased expression of pectin lyase in the mutant strain.

### 3.7 Measurement of transcript levels

Differences in the relative abundances of mRNAs were used to show transcript levels in the original strain versus those in the mutant strain. As shown in Supplementary Figure S7, the mRNA abundance in the mutant strain *E. coli* BL21/1G3 was increased 1.3-fold (*p* < 0.001) relative to that in the original strain *E. coli* BL21/PGLA-rep4. The mutant strain also exhibited a higher level of transcription and, therefore, relatively higher expression of PGLA-rep4.

## 4 Discussion

In the process of papermaking, the high temperature and highly alkaline environment result in a large amount of pectin substances being freed from the wood fibers, which not only affects the environmental quality, but also increases the production costs and decreases the paper quality. Samanta. (2019) applied pectinase in the papermaking process and found that it reduced the chemical and energy consumption and environmental stress, produced more uniform, soft, and shiny paper, and resulted in significantly higher paper strength than that obtained using the traditional soda ash cooking method, showing its good economic benefits. Therefore, the production of alkaline pectin lyase with high enzyme activity at a low cost is important to solve the ecological and economic problems of the pulp and paper industry (Saharan and Sharma, 2019; Zheng et al., 2021).

Differences in expression hosts affect the relative expression of enzymes. Compared to yeast, *E. coli* can efficiently receive (integrate) foreign DNA and express recombinant proteins at a very high rate (Kaur et al., 2018; Swain et al., 2023), which makes the heterologous expression of genes in *E. coli* much easier. As early as in the previous study, the *E. coli* vector was utilized to express PGLA and showed high enzyme activity (Zhou et al., 2017), which proved to be an excellent expression vector for PGLA. And by SDS-PAGE, it was found that the expression of PGLA in *P. pastoris* was low, and thus it was not suitable as an expression vector for PGLA. In this study, we obtained highly viable enzymes from *E. coli* expression strains after changing the expression host for PGLA-rep4.

Enzyme expression can be effectively increased through UV mutagenesis. UV irradiation causes two neighboring purine bases (adenine and guanine) in a DNA molecule to form a covalent bond, resulting in the formation of a purine dimer. This dimer results in structural changes in the DNA molecule, affecting protein expression and function (Ikehata and Ono, 2011). Via UV mutagenesis, we obtained a mutant strain with a mutation in the gene encoding ApaH and a base deletion in the gene encoding RNase E. The regulation of protein expression is divided into transcriptional and translational levels, where the efficiency of translation is directly related to mRNA degradation. RNase E converts and degrades mRNA (Morrison and Dunman, 2011; Chavez et al., 2016), and the deletion of bases in the gene encoding RNase E can lead to the erroneous accumulation of mRNA for a short period, which is detrimental to cell growth (Hughes, 2016). Additionally, this can diminish the effective protection of mRNA from the binding of more ribosomes, resulting in mRNA degradation. However, the redundant mRNA can continue to contribute to transient protein expression (Abreu et al., 2009; Tavernier et al., 2011). Meanwhile, the *ApaH* gene encodes a diadenosine tetraphosphatase that hydrolyzes 5′,5″-P1, P4 adenosine tetraphosphate (AppppA) to form two molecules of adenosine diphosphate (ADP), which is present in all living cells, from archaebacteria to humans, and has long been considered an intracellular alarm molecule in prokaryotes and eukaryotes. Within the cell, it affects biological processes, such as DNA repair, RNA programming, cell differentiation, thermal excitation and oxygen partial pressure, apoptosis, and transcriptional regulation (Ji et al., 2019). Mutations induced by base substitutions can enhance ApaH activity, and highly active ApaH, in turn, can lead to the entry of degraded N-RNAs of methylated Np_n_Ns into a state of rapid cell turnover (Hudecek et al., 2020), all of which can lead to the increased expression of PGLA-rep4.

In recent years, directed evolution has become a powerful tool for improving enzyme properties. Since the use of high-throughput sequencing and macrogenomic techniques allows rapid evaluation of mutant libraries, this technique has become the gold standard for microbiome analysis, but it is relatively time-consuming and does not allow determination of microbial activity turnover(Nakamura et al., 2011; Gray et al., 2015; El Mujtar et al., 2022). Therefore, it is important to construct an efficient screening method to analyze the mutant library of pectin lytic enzymes. Here, we demonstrated pectin lyase expression, based on the expression of fluorescent proteins, and performed the high-throughput screening of cells via flow cytometry, which resulted in a high-expression mutant strain.

In conclusion, the selection of high-vigor pectin lyase strains remains a key goal for the industry. We can maximize the solution to the problem of wastewater pollution in the paper industry only by continuously improving the enzyme activity, but further improving pectin lyase activity can be challenging. We constructed strains with higher pectin lysin enzyme activity that are more suitable for industrial applications by changing the expression host and UV mutagenesis, combined with high-throughput screening. Our results provide an effective method for screening high-vigor pectin lyase-producing strains and can be used for the expression of other enzymes.

## Data Availability

The datasets presented in this study can be found in online repositories. The names of the repository/repositories and accession number(s) can be found below: https://www.ncbi.nlm.nih.gov/, PRJNA988841.
