# Supplementary material for: Flow-cytometric cell sorting coupled with UV mutagenesis for improving pectin lyase expression
Source: Front Bioeng Biotechnol. 2023 Aug 31;11:1251342. doi: 10.3389/fbioe.2023.1251342 (PMC10502208; doi:10.3389/fbioe.2023.1251342)
Supplement: Supplementary file 1 [file DataSheet1.pdf]

Supplementary Material

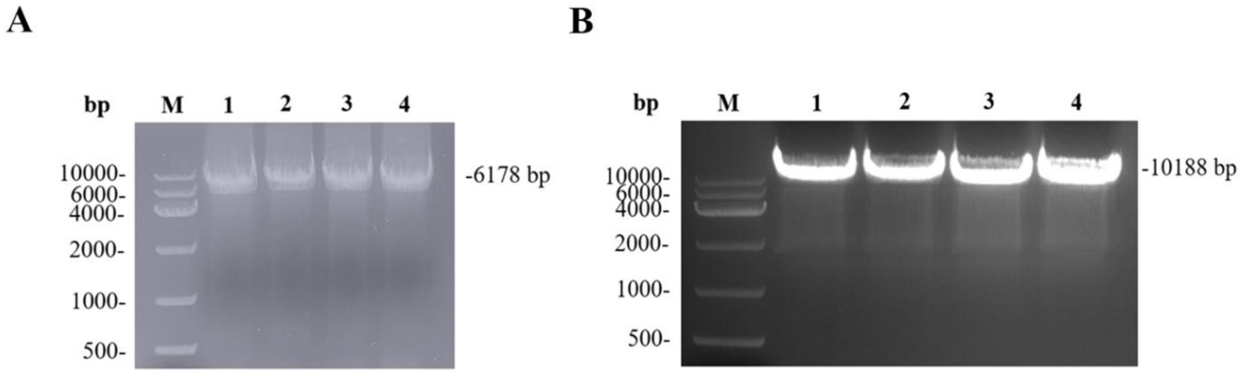

Fig. S1. Electrophoresis of DNA fragments. (A): Electrophoresis result of linearized plasmid pET28a-PGLA-rep4; (B): Electrophoresis result of linearized plasmid pPIC9K-PGLA-rep4.

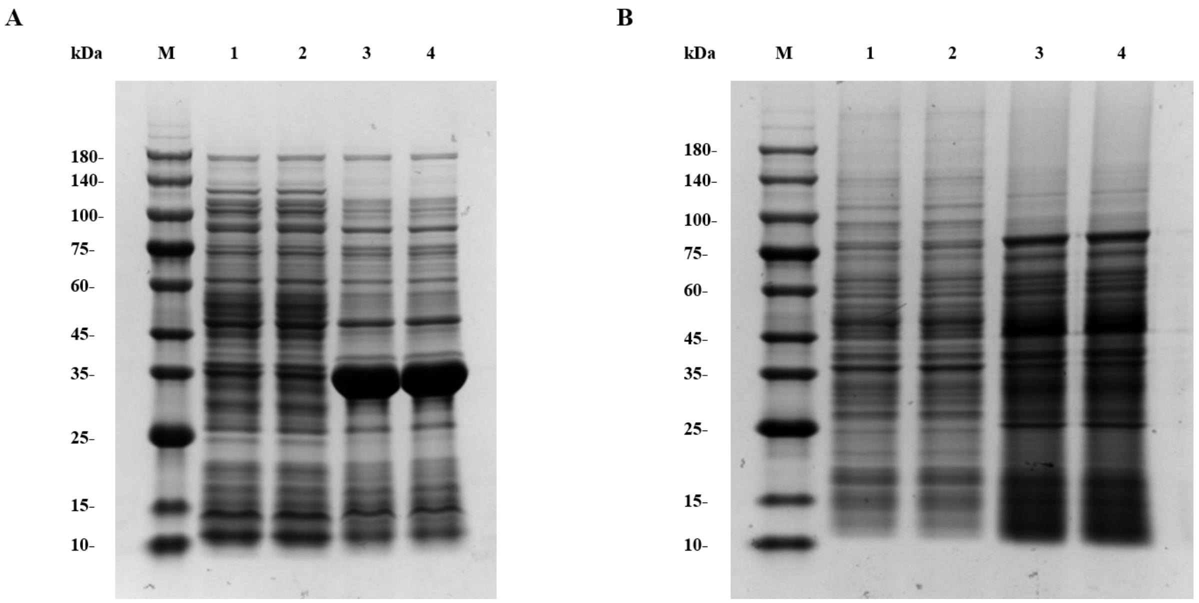

Fig. S2. SDS-PAGE analysis of PGLA-rep4 in *E. coli* and *P. pastoris* vectors. (A): SDS-PAGE analysis of PGLA-rep4 in the *E. coli* BL21. Lane M, molecular weight marker; Lane 1–2, E. coli blank control; Lane 3–4, protein electropherograms of BL21/ PGLA-rep4.; (B): SDS-PAGE analysis of PGLA-rep4 in the *P. pastoris* GS115 vector. Lane M, molecular weight labeling; Lane 1-2, P. pastoris blank control; Lane 3-4, protein electropherogram of GS115/PGLA-rep4.

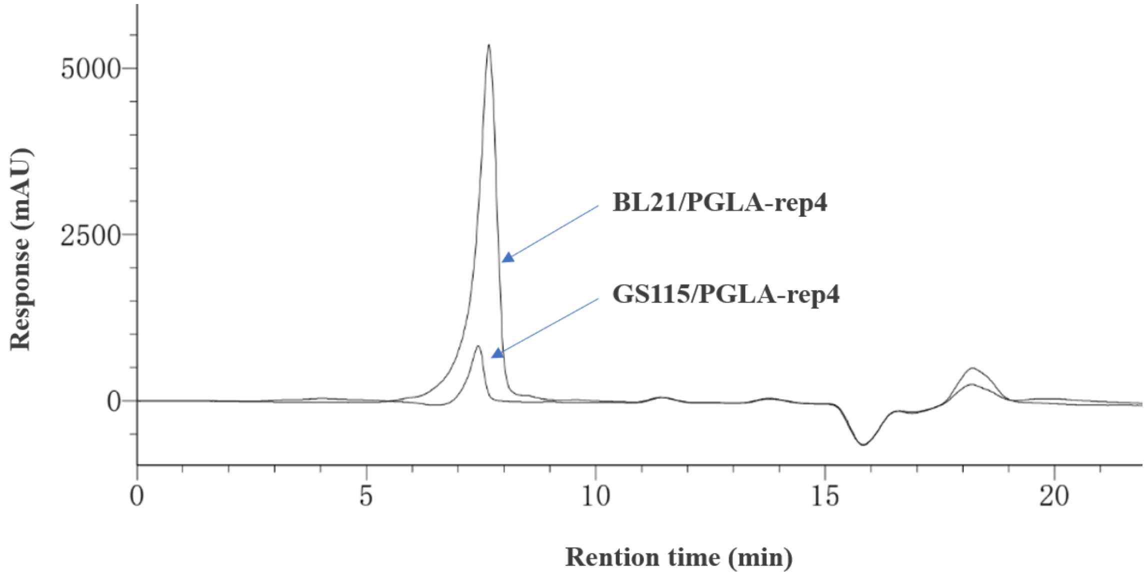

Fig. S3. The products of PGLA-rep4 hydrolysis of pectin were analyzed by high performance liquid chromatography (HPLC) after the reaction at 70°C for 10 min. The arrow show the position of galacturonic acid.

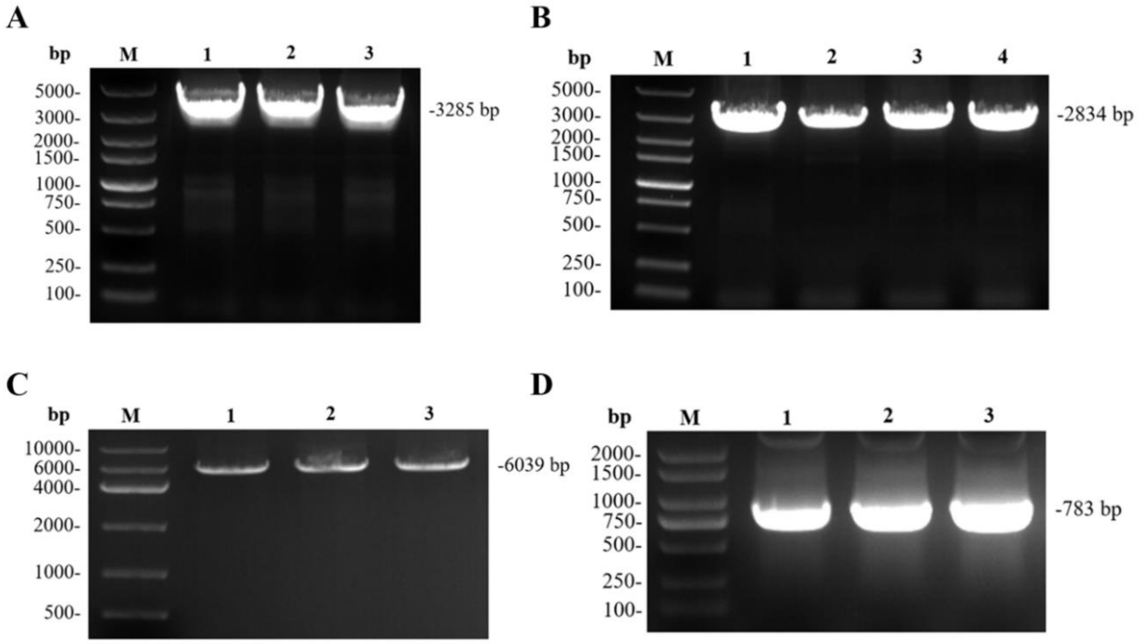

Fig. S4. Electrophoresis of DNA fragments. (A): Obtaining fragment containing temperature-sensitive gene on pKD46 by PCR reaction; (B): Obtaining fragments containing PGLA-rep4 gene, T7 promoter and lacI gene on pET28a-PGLA-rep4 by PCR reaction; (C): Nucleic acid electrophoresis of linearized plasmid pET28a-pKD46-PGLA-rep4; (D): Obtaining fragment containing egfp gene on pUC57-egfp by PCR reaction.

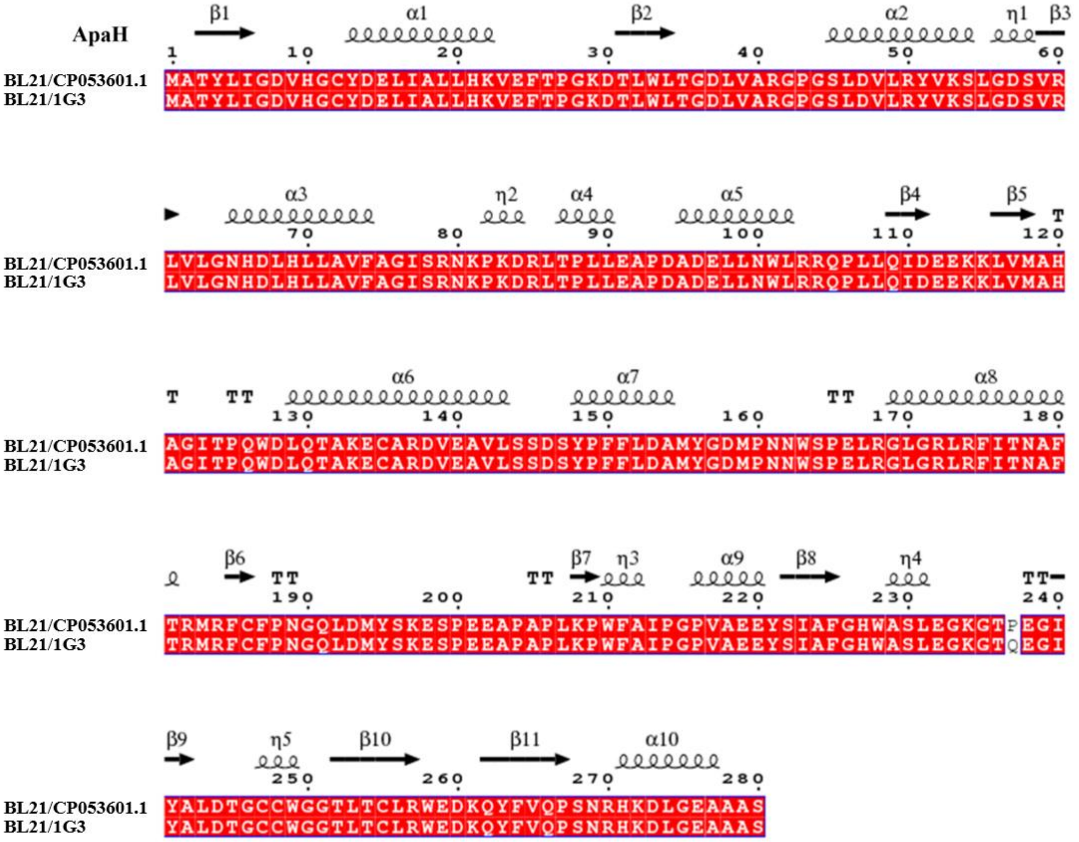

Fig. S5. Sequence comparison of base mutant strains with wild strains.

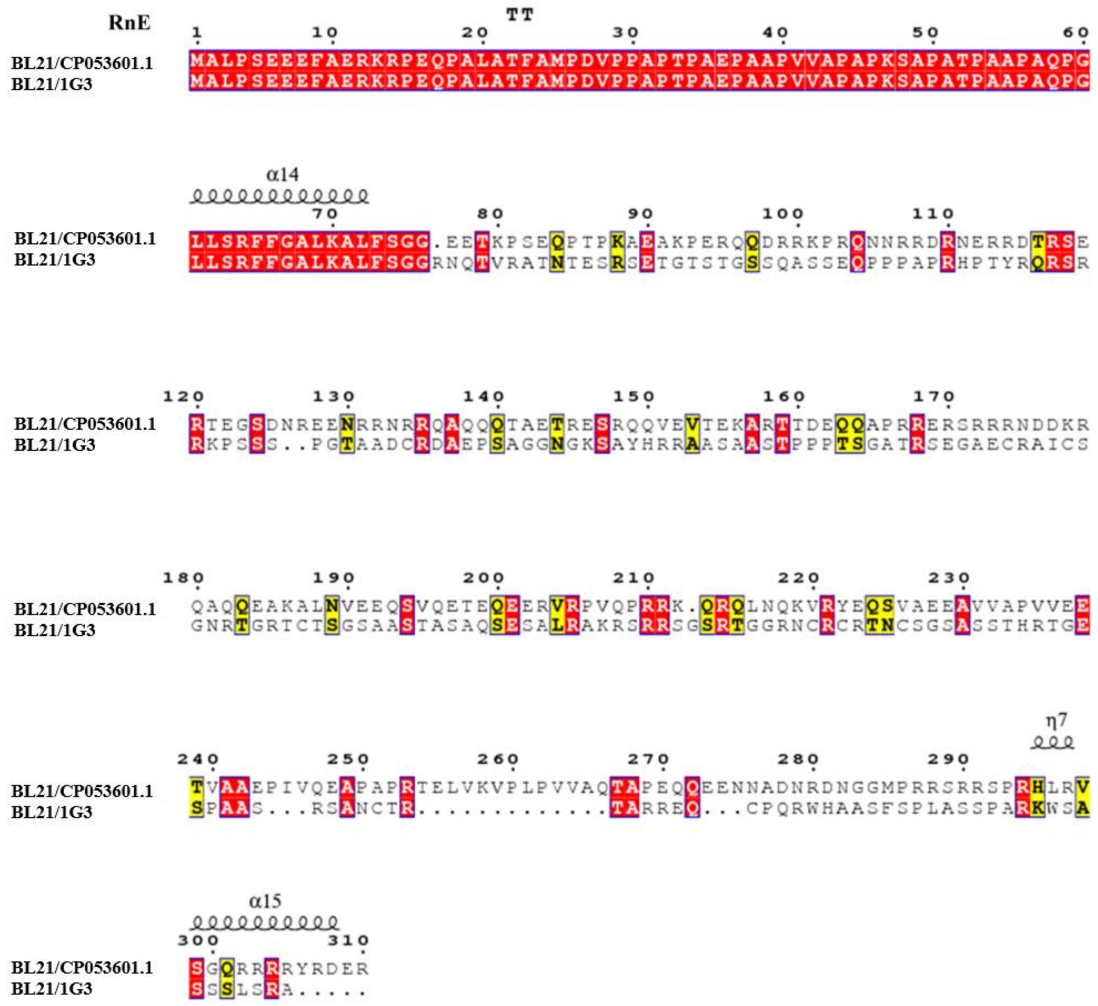

Fig. S6. Sequence comparison of base deletion strains with wild strains.

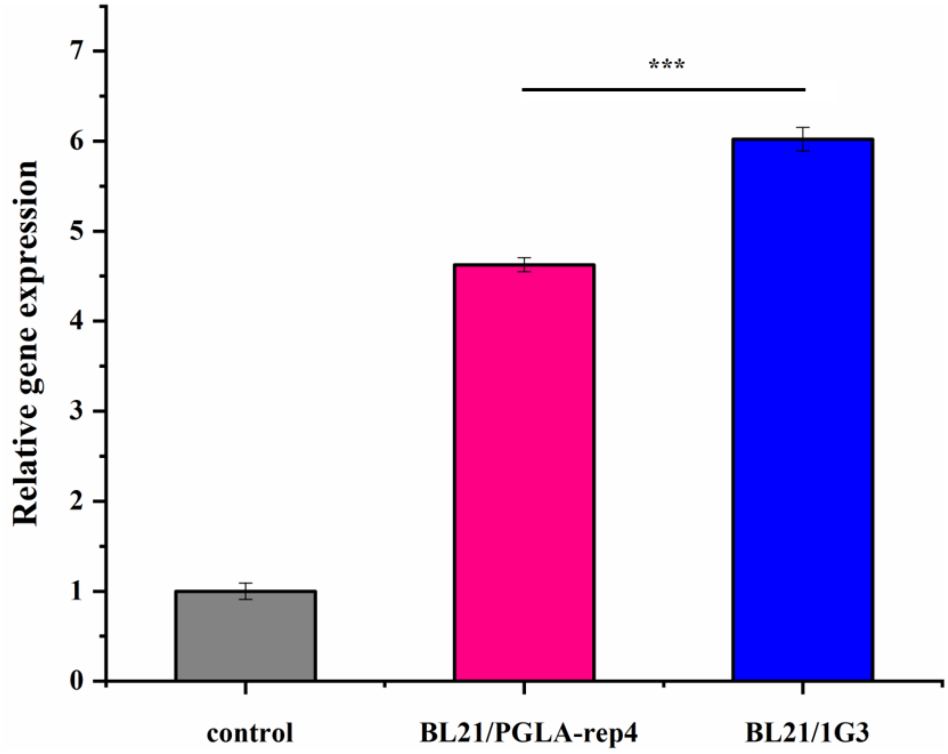

Fig. S7. Relative abundance of mRNA in the original strain and the mutant strain. Error bars represent standard deviations.
